# Efficacy of image-guided accurate limbal relaxing incisions for astigmatism correction during cataract surgery

**DOI:** 10.1016/j.aopr.2025.06.001

**Published:** 2025-06-04

**Authors:** Xiaoxin Hu, Jiao Qi, Kaiwen Cheng, Wenwen He, Yu Du, Keke Zhang, Yi Lu, Xiangjia Zhu

**Affiliations:** a Eye Institute and Department of Ophthalmology, Eye & ENT Hospital, Fudan University, Shanghai, China; b NHC Key Laboratory of Myopia and Related Eye Diseases, Key Laboratory of Myopia and Related Eye Diseases, Chinese Academy of Medical Sciences, Shanghai, China; c Shanghai Key Laboratory of Visual Impairment and Restoration, Shanghai, China

**Keywords:** Image-guided accurate limbal relaxing incisions, Cataract surgery, Astigmatism correction, Visual quality

## Abstract

**Purpose:**

To investigate the efficacy of image-guided accurate limbal relaxing incisions (LRIs) for astigmatism correction during cataract surgery.

**Methods:**

Consecutive cataract patients with regular corneal astigmatism ranging from 0.75 to 2.50 D, intended for cataract surgery with image-guided LRIs, were recruited in this prospective cohort study. The efficacy of astigmatism correction was evaluated 3 months after surgery, and compared among eyes with preoperative corneal with-the-rule (WTR), against-the-rule (ATR) and oblique astigmatism. Higher-order aberrations and visual quality indices obtained with iTrace were further compared between eyes with single and paired LRIs.

**Results:**

Totally, 108 eyes of 108 patients were analyzed. The mean total surgical induced astigmatism (tSIA) vector of all participants was 0.76 ​± ​0.38 D (range: 0.11–1.79 D, preoperative vs. postoperative astigmatism: 1.46 ​± ​0.41 vs. 0.78 ​± ​0.44 D, *P* ​< ​0.001). Eyes with WTR astigmatism showed higher tSIA (0.89 ​± ​0.32 D vs. 0.42 ​± ​0.21 D vs. 0.48 ​± ​0.36 D, respectively, *P* ​< ​0.001), as well as higher correction index and lower difference vector and index of success than ATR and oblique astigmatism groups (all *P* ​< ​0.05). Eyes with paired LRIs exhibited better corneal average height of modulation transfer function, a better corneal performance index and a better quality of vision index than those with single LRI (all *P* ​< ​0.05).

**Conclusions:**

Image-guided LRIs can effectively correct low-to-moderate corneal astigmatism during cataract surgery, especially in eyes with WTR astigmatism.

## 1 Introduction

With enhancements in phacoemulsification techniques and progress in intraocular lenses (IOLs), the anticipations and requirements of patients concerning cataract surgery are persistently escalating. Corneal astigmatism is among the most notable determinants impacting visual acuity subsequent to cataract surgery.^1^ Despite the occurrence of pre-existing corneal astigmatism in cataract patients, reports indicate that approximately 35%–47% of patients exhibit over 1.0 diopter (D) of corneal astigmatism, while 8%–13% have in excess of 2.0 D.^2^, ^3^, ^4^ Consequently, astigmatism management is vital for attenuating residual refractive error and attaining superior visual results. Presently, various techniques have been introduced to reduce corneal astigmatism during cataract surgery.

Limbal relaxing incisions (LRIs) are non-penetrating incisions made on the steep meridian of the corneal periphery for the treatment of low-to-moderate corneal astigmatism.^5^, ^6^, ^7^, ^8^ Compared with other techniques, like toric IOL implantation and femtosecond laser arcuate keratotomies, LRIs have the advantages in terms of cost-effectiveness and ease of operation, as it is done during the same procedure.^9^, ^10^, ^11^

However, the traditional manual LRIs face notable challenges regarding accuracy, repeatability and predictability, since these procedures heavily depend on the surgeons’ experience to determine the depth, length, angulation, alignment, and localization of the corneal incisions.^12^^,^^13^ For instance, improper placement and size of the incisions can induce irregular astigmatism if the corneal surface is altered unevenly.^12^ Inadequate incisions due to the imprecise procedures will result in insufficient astigmatism correction and dissatisfied visual acuity, failing to achieve the desired surgical outcomes.^13^

With the advancements in technology, many new techniques have been introduced to facilitate accurate LRIs. Firstly, LRI nomograms and online calculators have been developed and optimized to assist surgeons in designing precise plans for LRIs.^14^^,^^15^ Besides, numerous methodologies have been devised to manage corneal astigmatism during cataract surgery. Notably, intraoperative image-guided marking systems, such as Callisto eye, can simplify the preoperative manual marking process and improve the precision of incisions. By projecting real-time digital image guidance onto the eye, these systems enable accurate identification of the target incision location on the operating microscope.^12^^,^^16^ However, to our knowledge, there are no detailed evaluations of the effectiveness of image-guided accurate LRIs in managing low-to-moderate corneal astigmatism during cataract surgery.

## 2 Material and methods

### 2.1 Patients

This prospective study garnered approval from the Institutional Review Board of the Eye and Ear, Nose, and Throat (EENT) Hospital of Fudan University, Shanghai, China (No.2020068), adhering to the principles of the Declaration of Helsinki. Prior to cataract surgery, all participants provided signed informed consents authorizing the utilization of their clinical data. The study was affiliated with the Shanghai High Myopia Study initiated at the EENT Hospital of Fudan University in 2015 (www.clinicaltrials.gov, NCT03062085).

Consecutive cataract patients intending to undergo image-guided accurate LRIs during cataract surgery at the EENT Hospital of Fudan University from January 2021 to September 2023 were enrolled. Eligibility criteria comprised cataract patients aged ≥40 years, displaying regular and symmetric anterior corneal astigmatism on the corneal topographic map within the range of 0.75–2.5 D. Exclusion criteria encompassed: 1) irregular astigmatism (e.g., corneal scar, corneal degeneration, keratoconus); 2) severe dry eye; 3) previous ocular surgery or trauma; 4) other ocular conditions, such as strabismus, uveitis, glaucoma, severe retinal pathology; 5) substantial intraoperative or postoperative complications, including posterior capsule rupture or futile continuous circular capsulorhexis; and 6) patients lost to follow-up. In instances where both eyes of a patient satisfied the criteria, one eye was selected at random.

### 2.2 Preoperative examinations

Comprehensive ophthalmic assessments, including the evaluation of visual acuity, non-contact tonometry (NCT), IOLMaster 700 (Carl Zeiss AG, Jena, Germany), B-scan ultrasonography, corneal tomography (Pentacam HR; Oculus Optikgeräte GmbH, Wetzlar, Germany), slit-lamp microscopy, funduscopy, and image-forming coherence tomography (OCT, Zeiss Cirrus HD-OCT 5000; Carl Zeiss AG, Jena, Germany), were executed prior to surgery. The Barrett Universal II formula was employed for calculating IOL power. The postoperative digital reference images acquired during the examination with the IOLMaster 700, which included details of limbal and scleral vessels, were imported into the Callisto eye image-guided system (Carl Zeiss AG, Oberkochen, Germany) for real-time intraoperative eye tracking and precise matching. With-the-rule (WTR) and against-the-rule (ATR) astigmatism were characterized as having the steep corneal meridian measured by Pentacam within the range of 60–120° and either 0–30° or 150–180°, respectively, and oblique astigmatism was defined as having all values in between.

### 2.3 LRIs calculation

**Fig. 1:**
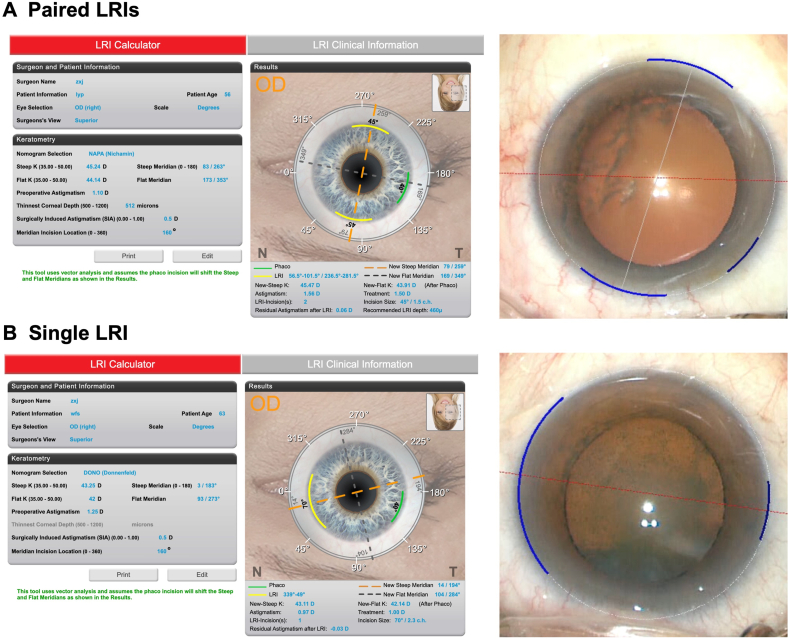
Examples of LRI online calculator and image-guided system. (A) An example of paired LRIs: Patient's keratometry and the main phacoemulsification laceration at 160° with an SIA of 0.5 D were inputted on the left. The recommended LRIs were shown in the middle, and the image-guided system used during surgery was exhibited on the right. The blue arc indicated the paired LRI-incisions and the main phacoemulsification incision. (B) An example of a single LRI. Patient's keratometry and the main phacoemulsification laceration at 160° with an SIA of 0.5D were inputted on the left. The recommended LRI was exhibited in the middle, and the image-guided system used during surgery was displayed on the right. The blue arc indicated the single LRI-laceration and main phacoemulsification incision.

The length and position of the LRIs were calculated using the online calculator (http://www. lricalculator.com) by inputting patients’ steep and flat keratometry measurements from Pentacam. The surgical induced astigmatism (SIA) vector was previously calculated as 0.5 D for the surgeon, which was used for the online calculation. The widely accepted system, Nichamin Age and Pachymetry-adjusted Intralimbal Arcuate Astigmatic (NAPA) calculator (Fig. 1A) was chosen to calculate paired LRIs. The main phaco laceration was positioned at 160° for all patients. If the results of NAPA calculator indicated that one of the LRIs and phacoemulsification laceration were overlapped, we further change to plan a single LRI using Donnenfeld (DONO) calculator (Fig. 1B).

### 2.4 Surgical technique

A single, proficient surgeon (Prof. Zhu XJ) performed all surgeries adhering to a standard procedure. The Callisto eye image-guided system was pre-programmed with calculation results, enabling an intraoperative overlay to be displayed under microscope for guiding the length and position of LRIs. Before initiating the clear corneal laceration process, LRIs were executed at 1 ​mm inside the limbus utilizing a 500 ​μm or 550 ​μm LRI knife (MLR50/MLR55; MANI; Japan), determined by patients' thinnest corneal thickness recorded pre-surgically using Pentacam. In cases with a thinnest corneal thickness exceeding 525 ​μm, the 550 ​μm blade was employed, while the 500 ​μm blade was reserved for instances exhibiting a thinnest corneal thickness of ≤525 ​μm. An example of the online LRI calculation and surgical procedure is presented in Supplementary data.

After LRIs were complete, conventional cataract surgery was performed. A 2.6 ​mm clear corneal incision was made for all patients as the main phacoemulsification incision. Subsequently, a 5.5 ​mm continuous curvilinear capsulorhexis, hydrodissection, and phacoemulsification ensued. The IOL (Zeiss CT ASPHINA 409 ​MP IOLs, Jena, Germany) was inserted into the capsular bag and aligned with the center. Once the viscoelastic material was entirely eradicated, the IOL position was recentered, followed by incision hydration.

Patients were instructed to take routine postoperative medications, involving topical prednisolone acetate (Allergan Pharmaceutical Ireland, Westport, Ireland), gatifloxacin ophthalmic drops (Otsuka Pharmaceutical, Tianjin, China), and pranoprofen ophthalmic drops (Pranopulin, Senju Pharmaceutical, Osaka, Japan), 3 times daily for 4 weeks.

### 2.5 Postoperative examinations

Comprehensive ophthalmic examinations were conducted three months after surgery. Uncorrected visual acuity (UCVA; logarithms of the minimal angle of resolution, logMAR) and best-corrected visual acuity (BCVA; logMAR) were evaluated. Assessments involving NCT, manifest refraction, and slit-lamp microscopy were recorded. The postoperative spherical equivalent (SE) of manifest refraction was calculated as sphere ​+ ​cylinder/2. The Simk value obtained from Pentacam was recorded as the postoperative corneal astigmatism.

All patients also underwent iTrace Visual Function Analyzer (iTrace, Tracey Technologies Corporation, Houston, TX, USA) assessments after pupil dilation. A specialized technician conducted these evaluations within a darkroom environment. The iTrace device combines Placido disk-based corneal topography and a ray tracing aberrometer,^17^ allowing for a comprehensive analysis of visual function. Initially, ocular aberration measurements were acquired via the ray tracing aberrometer. Subsequently, Placido rings were superimposed onto the corneal tear film, and an automated image capture was executed. Utilizing its integrated software and algorithms, the iTrace identified the ring edges and computed corneal curvature, refractive power, visual axes, and corneal wavefront data. With this collective information, the software calculated higher-order aberrations (HOAs), average height of modulation transfer function (aMTF), as well as the values of corneal performance index (CPI), and quality of vision index (QVI) – metrics derived from aberration data. Both corneal and ocular HOAs and aMTF for 4-mm and 6-mm pupil diameters were analyzed in this study. CPI, which assesses the image-forming quality of the cornea, and QVI, which quantifies the total image-forming performance of the whole eye, have been considered as novel objective visual quality parameters.^18^^,^^19^ These objective indexes values range from 0 (very poor) to 10 (excellent).

### 2.6 Statistical analysis

The vector analysis of astigmatic correction was performed using the Alpins method. The corneal astigmatism measurements obtained from Pentacam were used for calculation. Target induced astigmatism (TIA) is defined as the intended correction in astigmatic magnitude and axis. tSIA is the amount and axis of astigmatism change achieved by surgery, reflecting the combined impact of the surgical incision and the LRIs. Difference vector (DV) is the astigmatism magnitude and correction further required to achieve the intended outcome. The magnitude of error (ME) and the angle of error (AE) is the difference in magnitude and angle between the tSIA and the TIA, respectively. The correction index (CI) is the ratio of the tSIA to the TIA, and the index of success (IOS) is a measure of success with an ideal value of 0 and is calculated by the DV divided by the TIA.

Data analysis was accomplished using SPSS version 22 (IBM Corp., Chicago, IL, USA), and visual representations were crafted employing Prism 9 software (GraphPad, La Jolla, California, USA). Continuous variables are conveyed as mean ​± ​standard deviation, utilizing Student's *t*-test for comparing two groups and one-way analysis of variance (ANOVA) alongside Tukey's post hoc test for multiple group contrasts. Categorical data are expressed as frequency (percentage) and examined via the χ^2^ test. A P-value <0.05 served as the indicator for statistical significance.

## 3 Results

### 3.1 Patient characteristics

Table 1 shows the baseline characteristics of all the participants. A total of 108 eyes (60 right and 48 left) from 108 patients (50 men and 58 women) were included in the analysis. The pre-existing corneal astigmatism was 1.46 ​± ​0.42 D, ranging from 0.80 D to 2.48 D. Among all participants of this study, 74 eyes had WTR astigmatism, 26 eyes had ATR astigmatism, and 8 eyes had oblique astigmatism. Additionally, 27 eyes underwent a single LRI, while 81 eyes received paired LRIs. There was no statistical significance among eyes with WTR, ATR and oblique astigmatism in terms of age, sex, eye laterality, AL, anterior chamber depth, lens thickness, white-to-white distance, and preoperative corneal astigmatism, and the number of LRIs (one-way ANOVA with Tukey's post hoc test or *χ*^2^ test, all *P* ​> ​0.05).

**Table 1:** Preoperative patient characteristics.

	All participants	Types of corneal astigmatism			
		WTR (74 eyes)	ATR (26 eyes)	Oblique (8 eyes)	*P*-value
Age (years)	59.9 ​± ​9.4	58.5 ​± ​8.1	62.8 ​± ​12.7	62.8 ​± ​5.8	0.093
Sex (male/female)	50/58	29/45	16/10	5/3	0.092
Eye laterality (right/left)	60/48	45/29	12/14	3/5	0.204
Axial length (mm)	26.06 ​± ​2.09	26.31 ​± ​2.08	25.59 ​± ​2.22	25.26 ​± ​1.35	0.168
Anterior chamber depth (mm)	3.33 ​± ​0.39	3.36 ​± ​0.39	3.34 ​± ​0.39	3.08 ​± ​0.31	0.166
Lens thickness (mm)	4.38 ​± ​0.43	4.40 ​± ​0.41	4.35 ​± ​0.45	4.29 ​± ​0.58	0.695
White-to-white distance (mm)	12.10 ​± ​0.32	12.13 ​± ​0.33	11.99 ​± ​0.27	12.13 ​± ​0.32	0.149
Pre-UDVA (logMAR)	0.75 ​± ​0.39	0.76 ​± ​0.42	0.73 ​± ​0.35	0.77 ​± ​0.36	0.957
Pre-CDVA (logMAR)	0.63 ​± ​0.46	0.70 ​± ​0.41	0.61 ​± ​0.58	0.59 ​± ​0.40	0.509
Pre-corneal astigmatism (D)	1.46 ​± ​0.41	1.49 ​± ​0.41	1.39 ​± ​0.40	1.38 ​± ​0.41	0.527
Number of LRIs					0.243
Single LRI	27 (25.00%)	15	9	3	
Paired LRIs	81 (75.00%)	59	17	5	

UDVA, uncorrected distance visual acuity; CDVA, corrected distance visual acuity; logMAR, logarithm of the minimal angle of resolution; WTR, with-the-rule; ATR, against-the-rule; LRI, limbal relaxing incision. Data were presented as mean ​± ​standard deviation.

### 3.2 Outcome of corneal astigmatism correction at 3 months after surgery

**Fig. 2:**
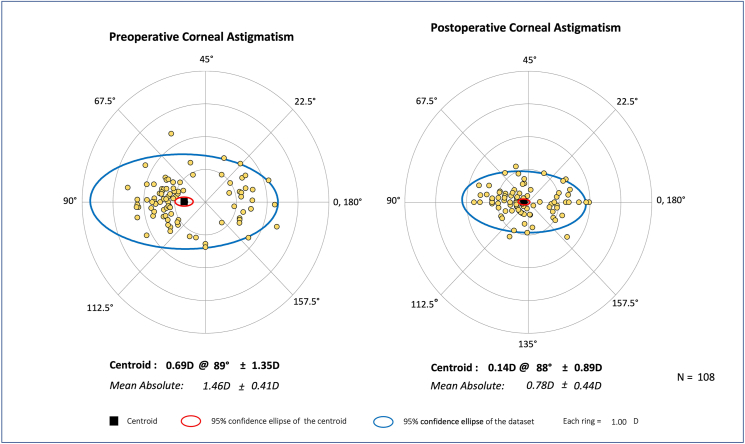
Double-angle plots of preoperative and postoperative corneal astigmatism of all participants at 3 months after surgery. Preoperative corneal astigmatism (left) and postoperative corneal astigmatism (right).

The distribution of preoperative and postoperative corneal astigmatism of all participants is shown in double-angle plots (Fig. 2). At 3 months after surgery, the mean postoperative corneal astigmatism was significantly reduced compared with their preoperative values (0.78 ​± ​0.44 D vs. 1.46 ​± ​0.41 D, Student's *t*-test, *P* ​< ​0.001). Notable improvements were also observed in both UCVA (0.39 ​± ​0.42 D vs. 0.75 ​± ​0.39 D, Student's *t*-test, *P* ​< ​0.001) and BCVA (0.06 ​± ​0.12 D vs. 0.63 ​± ​0.46 D, Student's *t*-test, *P* ​< ​0.001) relative to their preoperative values. Table 2 provides vector analysis values for all participants at 3 months post-surgery. The mean tSIA was 0.76 ​± ​0.38 D, ranging from 0.11 D to 1.79 D, among which 81 (75.00%) eyes experienced an astigmatic correction of more than 0.5 D and 29 eyes (26.85%) had a correction exceeding 1.0 D.

**Table 2:** Refractive outcomes at 3 months after surgery.

	All participants	Types of corneal astigmatism			
		WTR (74 eyes)	ATR (26 eyes)	Oblique (8 eyes)	*P*-value
UCVA (logMAR)	0.39 ​± ​0.42	0.31 ​± ​0.40	0.57 ​± ​0.42	0.55 ​± ​0.43	0.015∗
BCVA (logMAR)	0.06 ​± ​0.12	0.05 ​± ​0.13	0.10 ​± ​0.10	0.08 ​± ​0.03	0.163
SE (D)	−1.38 ​± ​1.65	−1.29 ​± ​1.43	−1.72 ​± ​1.61	−1.42 ​± ​1.61	0.521
Postoperative refractive sphere (D)	−1.14 ​± ​1.42	−1.10 ​± ​1.44	−1.35 ​± ​1.23	−0.98 ​± ​1.69	0.603
Postoperative refractive cylinder (D)	0.71 ​± ​0.38	0.60 ​± ​0.33	0.97 ​± ​0.37	0.92 ​± ​0.29	< 0.001∗
TIA (D)	1.44 ​± ​0.47	1.48 ​± ​0.41	1.35 ​± ​0.44	1.37 ​± ​0.94	0.696
tSIA (D)	0.76 ​± ​0.38	0.89 ​± ​0.32	0.42 ​± ​0.21	0.48 ​± ​0.36	< 0.001∗
DV (D)	0.81 ​± ​0.45	0.72 ​± ​0.40	1.04 ​± ​0.43	0.93 ​± ​0.72	0.016∗
ME (D)	−0.65 ​± ​0.53	−0.59 ​± ​0.46	−0.93 ​± ​0.48	−0.89 ​± ​0.72	0.019∗
AE (°)	2.33 ​± ​5.64	2.11 ​± ​5.22	2.76 ​± ​6.62	3.04 ​± ​6.63	0.862
CI	0.57 ​± ​0.27	0.65 ​± ​0.25	0.37 ​± ​0.22	0.40 ​± ​0.26	< 0.001∗
IOS	0.57 ​± ​0.22	0.49 ​± ​0.18	0.77 ​± ​0.14	0.69 ​± ​0.34	< 0.001∗

UCVA, uncorrected visual acuity; BCVA, best corrected visual acuity; logMAR, logarithm of the minimal angle of resolution; SE, spherical equivalent; D, diopter; TIA, target induced astigmatism; tSIA, total surgically induced astigmatism; DV, difference vector; ME, magnitude of error; AE, angle of error; CI, correction index; IOS, index of success; WTR, with-the-rule; ATR, against-the-rule. Data were presented as mean ​± ​standard deviation. ∗Statistically significant (*P* ​< ​0.05) values.

### 3.3 Differences in corneal astigmatism correction among various types of corneal astigmatism

**Fig. 3:**
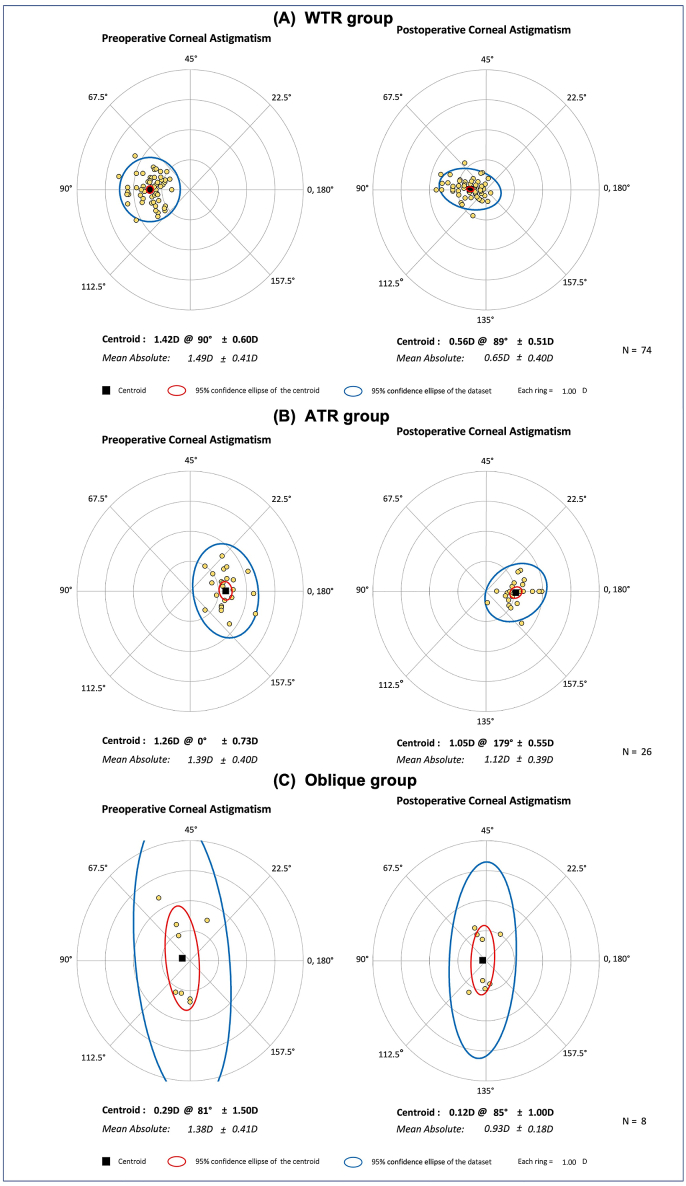
**Double-angle plots of preoperative and postoperative corneal astigmatism of eyes with WTR, ATR and oblique astigmatism at 3 months after surgery.** (A) Preoperative corneal astigmatism (left) and postoperative corneal astigmatism (right) of eyes with WTR astigmatism. (B) Preoperative corneal astigmatism (left) and postoperative corneal astigmatism (right) of eyes with ATR astigmatism. (C) Preoperative corneal astigmatism (left) and postoperative corneal astigmatism (right) of eyes with oblique astigmatism. WTR, with-the-rule; ATR, against-the-rule.

The distribution of preoperative and postoperative corneal astigmatism of eyes with WTR, ATR and oblique astigmatism are shown in Fig. 3. At 3 months after surgery, the mean postoperative corneal astigmatism of WTR group was significantly lower the other two groups (0.65 ​± ​0.40 D vs. 1.12 ​± ​0.39 D vs. 0.93 ​± ​0.18 D, one-way ANOVA with Tukey's post hoc test, *P* ​< ​0.001). The WTR astigmatism group presented significantly lower postoperative refractive cylinder than the ATR and oblique astigmatism group (0.60 ​± ​0.33 D vs. 0.97 ​± ​0.37 D vs. 0.92 ​± ​0.29 D, one-way ANOVA with Tukey's post hoc test, *P* ​< ​0.001; Table 2). The WTR astigmatism group also presented significantly better UCVA than the ATR and oblique astigmatism group (0.31 ​± ​0.40 logMAR vs. 0.57 ​± ​0.42 logMAR vs. 0.55 ​± ​0.43 logMAR, one-way ANOVA with Tukey's post hoc test, *P* ​= ​0.015; Table 2). There was no statistical difference for BCVA among three groups (one-way ANOVA with Tukey's post hoc test, *P* ​= ​0.163; Table 2).

Table 2 also exhibts the vector analysis outcomes of various types of corneal astigmatism. The TIA of every group had no statistic difference. The mean tSIA in WTR astigmatism was significantly greater than that in ATR and oblique astigmatism (0.89 ​± ​0.32 D vs. 0.42 ​± ​0.21 D vs. 0.48 ​± ​0.36 D, one-way ANOVA with Tukey's post hoc test, *P* ​< ​0.001). The highest proportion of tSIA in the WTR astigmatism group was found in 0.51–1.00 D (56.76%), with 69 (93.24%) eyes corrected by more than 0.5 D and among which 27 eyes (36.49%) corrected by more than 1.0 D. While, the highest proportion of tSIA in the ATR and oblique astigmatism group were both found in the ≤ 0.50 D range (65.38% and 62.50%). Furthermore, the ME in WTR astigmatism was closest to 0 among the three groups (*P* ​< ​0.05). The WTR astigmatism group displayed significantly higher CI, and lower DV and IOS values compared to the other groups (all *P* ​< ​0.05). No statistically significant differences in AE was observed among the three groups.

### 3.4 Comparisons of visual quality between single and paired LRIs

**Fig. 4:**
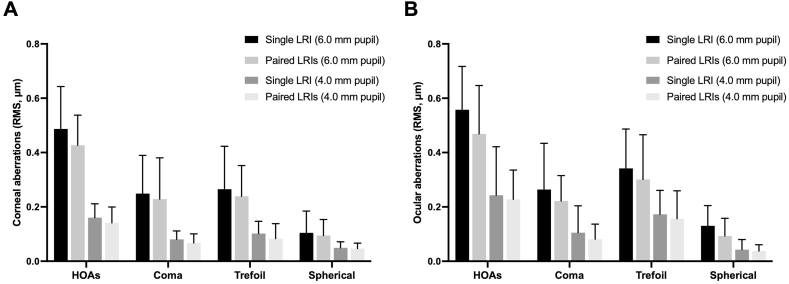
**Comparison of higher-order aberrations (HOAs) of eyes with single and paired LRIs at 3 months after surgery.** Corneal HOAs measured at pupil diameters of 6.0 ​mm and 4.0 ​mm ​(A) and ocular HOAs measured at pupil diameters of 6.0 ​mm and 4.0 ​mm ​(B). HOAs, higher-order aberrations; RMS, root mean square; LRI, limbal relaxing incision. Error bars represent the standard error of the mean.

The HOAs of the eyes with single and paired LRIs at 3 months after surgery are presented in Fig. 4. Regardless of whether measured at pupil diameters of 6.0 ​mm or 4.0 ​mm, eyes with paired LRIs showed slightly lower levels of corneal and ocular HOAs, coma, trefoil, and spherical aberrations compared to eyes with single LRI, although there was no statistical difference (Student's *t*-test, all *P* ​> ​0.05).

Contrasts between the eyes with single and paired LRIs in terms of postoperative visual quality indices are shown in Table 3. Eyes with paired LRIs showed a significantly higher value of corneal aMTF for 6 ​mm pupil diameter than those with single LRI (0.38 ​± ​0.08 D vs. 0.35 ​± ​0.07 D, Student's *t*-test, *P* ​= ​0.023), as well as s slightly higher value of corneal aMTF for 4 ​mm pupil diameter, though not statistically different (Student's *t*-test, *P* ​> ​0.05). Additionally, paired LRIs had significantly better CPI (8.31 ​± ​1.02 vs. 7.78 ​± ​0.87, Student's t-test, *P* ​= ​0.030) and QVI (8.12 ​± ​0.93 vs. 7.63 ​± ​0.72, Student's *t*-test, *P* ​= ​0.037) values compared to single LRI.

**Table 3:** Visual quality indices of eyes with single and paired LRIs at 3 months after surgery.

	Single LRI (27 eyes)	Paired LRIs (81 eyes)	*P*-value
Corneal aMTF (6.0-mm pupil diameter)	0.35 ​± ​0.07	0.38 ​± ​0.08	0.023∗
Ocular aMTF (6.0-mm pupil diameter)	0.34 ​± ​0.09	0.37 ​± ​0.09	0.128
Corneal aMTF (4.0-mm pupil diameter)	0.49 ​± ​0.10	0.51 ​± ​0.09	0.230
Ocular aMTF (4.0-mm pupil diameter)	0.46 ​± ​0.12	0.48 ​± ​0.10	0.334
CPI	7.78 ​± ​0.87	8.31 ​± ​1.02	0.030∗
QVI	7.63 ​± ​0.72	8.12 ​± ​0.93	0.037∗

aMTF, average height of modulation transfer function; CPI, corneal performance index; QVI, quality of vision index; LRI, limbal relaxing incision. Data were presented as mean ​± ​standard deviation. ∗Statistically significant (*P* ​< ​0.05) values.

## 4 Discussion

Contemporary cataract surgery has transformed into a refractive practice, augmenting anticipations for improved visual clarity, enhanced visual quality, and reduced reliance on spectacles. Consequently, more attention has been paid to the necessity of astigmatism correction during cataract surgery.

LRIs are used for correcting low-to-moderate regular astigmatism during cataract surgery. However, traditional manual LRIs raise Notable concerns due to their relatively poor precision and predictability.^9^^,^^20^ Image-guided LRIs, propelled by advanced real-time digital guidance technology, are anticipated to achieve relative accurate astigmatism correction and good postoperative visual quality.^21^^,^^22^ Therefore, in this study, we evaluated the efficacy of image-guided accurate LRIs in correcting low-to-moderate regular corneal astigmatism during cataract surgery in details.

In this study, we included patients with pre-existing anterior corneal astigmatism ranging from 0.75 D to 2.5 D. Postoperatively, their mean corneal astigmatism was significantly reduced, with most patients achieving a astigmatic correction of over 0.5 D. These outcomes confirm the effectiveness of image-guided LRIs. With precise pre-surgical calculation and design for LRI, combined with real-time intraoperative guidance, image-guided LRIs can provide predictable and satisfactory correction of corneal astigmatism. Besides, all eyes included in our study did not have any intraoperative or postoperative complications due to the procedure of LRIs. LRIs have been considered to be a relatively safe surgical technique for the treatment of corneal astigmatism during cataract surgery, because of its distance from the visual axis.^6^^,^^23^

Meanwhile, compared to traditional LRIs, image-guided LRIs offer numerous advantages. Firstly, it can simplify the surgical procedure by eliminating the need for pre-surgical marking, without extending the operating time. Conventional preoperative manual marking with an ink pen and slit-lamp is not only time-consuming and inconvenient but also lacks precision and reproducibility. Secondly, this approach can greatly alleviate patients’ psychological and ocular discomfort by avoiding contact with their eyes during marking. Thirdly, the use of real-time image-guided system during operation, in conjunction with online LRI calculators and calculators, ensures precise corneal incisions with accurate localization, length and depth. Furthermore, compared with femtosecond laser arcuate keratotomies, LRIs are more affordable and accessible. Thus, our study indicated that image-guided LRIs are an effective, safe, precise and cost-effective technique for addressing low-to-moderate regular corneal astigmatism.

Besides, our study demonstrated that LRIs performed for eyes with WTR astigmatism had a greater astigmatic correction effect compared to eyes with ATR and oblique astigmatism. Similar results have been reported by Chiam et al.^24^ and Lim et al.^25^ The following could be speculated as possible reasons. Primarily, vertical LRIs for WTR astigmatism may impede the healing of corneal incisions and tend to gape, due to the mechanical stress from intraocular pressure and eye blink, potentially delaying the reduction in the flattening impact of LRIs over time as a result of laceration healing.^26^^,^^27^ Furthermore, the horizontal cornea diameter is approximately 1.0 ​mm greater than the vertical diameter geometrically.^28^ Therefore, the horizontal LRIs are farther from the center of the cornea compared to vertical LRIs, which suggest a lower correction effect.^29^^,^^30^ Additionally, synchrotron x-ray diffraction reveals that the collagen fibrils near the limbus is oriented in superior-inferior and nasal-temporal directions, with 45° and 90° bends, respectively.^31^, ^32^, ^33^ Consequently, LRIs along the horizontal meridian would minimize the correction effect since they are parallel to most of the collagen fibrils, while those along the vertical meridian are likely to sever more fibrils, resulting in a greater flattening effect of the cornea.

In our study, eyes with paired LRIs exhibited significantly better corneal aMTF for a 6 ​mm pupil diameter, and better values of CPI and QVI compared to those with single LRI. CPI and QVI, obtained from iTrace visual function analyzer, serve as novel objective metrics that assess the function of corneal and total image-forming performance by integrating multiple measurements, including HOAs, contrast sensitivity, and image-forming alignment. Thus, our study indicated that eyes with symmetrical paired LRIs can achieve better visual quality than those with asymmetrical single LRI, especially under conditions where the pupil is relatively large. This may be because paired incisions, compared to single incision, enhance the flattening effect on the steep meridian by creating a more evenly distributed and symmetrical change to the periphery corneal. This helps to increase structural integrity of the cornea, resulting in reduced visual distortions and improved visual outcome.^34^^,^^35^

## 5 Conclusions

In conclusion, our study indicated that image-guided LRI, as a precise and cost-effective technique, can effectively correct low-to-moderate regular corneal astigmatism during cataract surgery. LRI performed on patients with preoperative WTR corneal astigmatism can achieve more effective astigmatism correction, and eyes with paired LRIs can obtain better visual quality.

## Study approval

The authors confirm that any aspect of the work covered in this manuscript that involved human patients was conducted with the ethical approval of all relevant bodies and the study was performed in accordance with the Declaration of Helsinki, and the protocol was approved by the Ethics Committee of the Eye and Ear, Nose, and Throat (EENT) Hospital of Fudan University, Shanghai, China (No.2020068).

## Author contributions

The authors confirm contribution to the paper as follows: XZ and YL revised the manuscript, obtained funding, and supervised the process. XH, JQ and KC collected, analyzed the patient data and wrote the manuscript. WH and YD assisted in drafting the manuscript and revised the manuscript. KZ read and revised the manuscript. XH, JQ and KC contributed equally to this work. All authors reviewed the results and approved the final version of the manuscript.

## Funding

This article was supported by research grants from the National Key Research and Development Program of China, China (2022YFC2502800), National Natural Science Foundation of China, China (82271069, 82371040, 82122017, 81870642, 81970780, 81470613 and 81670835), Special Project of Shanghai Public Health Research, China (2024GKQ36), Science and Technology Innovation Action Plan of Shanghai Science and Technology Commission, China (23Y11909800), Outstanding Youth Medical Talents of Shanghai "Rising Stars of Medical Talents" Youth Development Program, Shanghai Municipal Health Commission Project, China (2024ZZ1025 and 20244Z0015).

## Declaration of competing interest

The authors declare that they have no known competing financial interests or personal relationships that could have appeared to influence the work reported in this paper.
